# Two-Layer Tight Frame Sparsifying Model for Compressed Sensing Magnetic Resonance Imaging

**DOI:** 10.1155/2016/2860643

**Published:** 2016-09-25

**Authors:** Shanshan Wang, Jianbo Liu, Xi Peng, Pei Dong, Qiegen Liu, Dong Liang

**Affiliations:** ^1^Paul C. Lauterbur Research Center for Biomedical Imaging, Shenzhen Institutes of Advanced Technology, Shenzhen, Guangdong 518055, China; ^2^The Beijing Center for Mathematics and Information Interdisciplinary Sciences, Beijing 100048, China; ^3^Biomedical and Multimedia Information Technology (BMIT) Research Group, School of Information Technologies, The University of Sydney, Sydney, NSW 2006, Australia; ^4^Nanchang University, Nanchang, Jiangxi, China

## Abstract

Compressed sensing magnetic resonance imaging (CSMRI) employs image sparsity to reconstruct MR images from incoherently undersampled K-space data. Existing CSMRI approaches have exploited analysis transform, synthesis dictionary, and their variants to trigger image sparsity. Nevertheless, the accuracy, efficiency, or acceleration rate of existing CSMRI methods can still be improved due to either lack of adaptability, high complexity of the training, or insufficient sparsity promotion. To properly balance the three factors, this paper proposes a two-layer tight frame sparsifying (TRIMS) model for CSMRI by sparsifying the image with a product of a fixed tight frame and an adaptively learned tight frame. The two-layer sparsifying and adaptive learning nature of TRIMS has enabled accurate MR reconstruction from highly undersampled data with efficiency. To solve the reconstruction problem, a three-level Bregman numerical algorithm is developed. The proposed approach has been compared to three state-of-the-art methods over scanned physical phantom and in vivo MR datasets and encouraging performances have been achieved.

## 1. Introduction

Compressed sensing magnetic resonance imaging (CSMRI) is a very popular signal processing based technique for accelerating MRI scan. Different from the classical fixed-rate sampling dogma Shannon-Nyquist sampling theorem, CS exploits the sparsity of an MR image and allows CSMRI to recover MR images from less incoherently sampled K-space data [1]. The classical formulation of CSMRI can be written as

(1) minu Wu1s.t. Fpu=f,

where *u* ∈ *𝒞* ^*Q*×1^ and *f* ∈ *𝒞* ^*P*×1^, respectively, denote the MR image and its corresponding undersampled raw K-space data, *F*_*p*_ ∈ *𝒞* ^*P*×*Q*^ represents the undersampled Fourier encoding matrix with *P* ≪ *Q*, and ‖*Wu*‖_1_ is an analysis model which sparsifies the image with transform *W* ∈ *𝒞* ^*Q*×*Q*^ under the *ℓ*_1_ norm constraint. *P* and *Q* are the number of image pixels and measured data. The classical formulation is typically equipped with total variation and wavelet and it can be solved very efficiently [1]. However, the efficiency comes at the expense of accuracy, especially with highly undersampled noisy measurements, due to lack of adaptability or insufficient sparsity promotion. To address this issue, there have been diverse methods proposed [2, 3] and we focus on the following three representative directions.

One main endeavor is employing nonlocal operations or redundant transforms to analytically sparsify the MR image [4]. Typical examples include nonlocal total variation regularization [5], patch-based directional wavelet [6], and wavelet tree sparsity based CSMRI techniques [7]. These methods generally have straightforward models; nevertheless, the reconstruction accuracy is not that perfectly satisfying due to lack of adaptability. We proposed one-layer data-driven tight frame DDTF for undersampled image reconstruction [8]. It is generally very efficient. But its performance is still limited due to its insufficient sparsity promotion and reliance on the Bregman iteration technique for bringing back the image details.

The other effort is training adaptive dictionary to sparsely represent the MR image in the synthesis manner. For example, DLMRI [9], BPFA triggered MR reconstruction [10], and our proposed TBMDU [3] employ dictionary learning to adaptively capture image structures while promoting sparsity. These methods can generally achieve accurate MR image reconstruction with strong noise suppression capability. Unfortunately, the complexity of these approaches is very high and the sparsity is still directly limited to one-layer representation of the target image.

The third group endeavors could be regarded as the variants of the above two efforts, which target employing the advantages of both the analysis and synthesis sparse models. For example, the balanced tight frame model [11] introduces a penalty term to bridge the gap between the analysis and synthesis model. Unfortunately, although it possesses a fascinating mathematical explanation, the sparsity promotion is still limited to a single layer and therefore its performance is only comparable to the analysis one. To further promote sparsity, a wavelet driven dictionary learning (named WaveDLMRI) [12] technique and our proposed total variation driven dictionary learning approach (named GradDLRec) [13] adaptively represent the sparse coefficients derived from the analysis transform rather than directly encode the underlying image. Nevertheless, despite achieving encouraging performances, they still rely on the computationally expensive dictionary learning technique.

Recently, there are double sparsity model and doubly sparse transforms proposed in general image/signal processing community [14, 15]. The double sparsity model tries to train a sparse dictionary over a fixed base, while the doubly sparse transform is devoted to learning an adaptive sparse matrix over an analytic transform. There is no doubt that their application to image denoising has presented promising results, albeit the two-layer sparsifying model is more concerned to assist efficient learning, storage, and implementation by constraining the dictionary sparse rather than focus on further triggering of the sparsity of the image.

Motivated by the above observations, we try to develop a two-layer tight frame sparsifying (TRIMS) model for CSMRI by sparsifying the image with a product of a fixed tight frame and an adaptive learned tight frame. The proposed TRIMS has several merits: (1) the tight frame satisfies the perfect reconstruction property which ensures the given signal can be perfectly represented by its canonical expansion [16]; (2) a tight frame can be implemented very efficiently since it satisfies *W* ^*H*^ *W* = *I*; (3) the adaptability has been kept by the second-layer tight frame tailored for the target reconstruction task; (4) the two-layer tight frame has enabled the image sparsity to be explored more sufficiently compared to the one-layer one. Furthermore, the two-layer tight frame also has a convolutional explanation, which extracts appropriate image characteristics to constrain MR image reconstruction [17]. We have compared our method with three state-of-the-art approaches of the above three directions, namely, DDTF-MRI, DLMRI, and GradDLRec on an in vivo complex valued MR dataset. The results have advised the proposed method could properly balance the efficiency, accuracy, and acceleration factors.

## 2. Theory

### 2.1. TRIMS Model

To reconstruct MR images from undersampled data, we propose a TRIMS model which can be implicitly described as

(2) $$\begin{array}{c} \underset{u,W_{b}\in ⋀}{\min}{\left\|\;F_{p}u-f\;\right\|}_{2}^{2}+\alpha {\left\|\;W_{b}\left(\;W_{a}u\;\right)\;\right\|}_{1}, \end{array}$$

where *W*_*a*_ is the fixed tight frame and *W*_*b*_ denotes the data-driven tight frame. ⋀ means the tight frame system, since a tight frame can be formulated with a set of filters under the unitary extension principle (UEP) condition [16]. The proposed model also has another approximately equivalent convolutional expression, which we name the explicit model

(3) $$\begin{array}{c} \underset{u,b_{m}}{\min}{\left\|\;F_{p}u-f\;\right\|}_{2}^{2}+\alpha \underset{m}{\sum }\;\underset{n}{\sum }{\left\|\;b_{m}*\left(\;a_{n}*u\;\right)\;\right\|}_{1}, \end{array}$$

where *a*_*n*_ are the fixed kernels and *b*_*m*_ denote the to-be-learned adaptive kernels.

### 2.2. TRIMS Algorithm

To solve the proposed model, we develop a three-level Bregman iteration numerical algorithm. Introducing a Bregman parameter *c*, we have the first-level Bregman iteration

(4) uk+1,Wbk+1=argminu,Wb∈⋀⁡Fpu−f+ck22+αWbWau1,ck+1=ck+Fpuk+1−f.

To attack the first subproblem in (4), we introduce an assistant variable *u*_*a*_ = *W*_*a*_ *u* and obtain the second-level iteration

(5) uak+1,Wbk+1=argminua,Wb⁡ μWau−ua+dk22+αWbua1,uk+1=argminu⁡Fpu−f+ck22+μWau−uak+1+dk22,dk+1=dk+Wauk+1−uak+1.

The subproblem regarding the update of *u* is a simple least squares problem admitting an analytical solution. Its solution satisfies the following normal equation:

(6) $$\begin{array}{c} F_{p}^{H}\left(\;F_{p}u-f+c^{k}\;\right)+\mu W_{a}^{H}\left(\;W_{a}u-u_{a}^{k}+d^{k}\;\right)=0. \end{array}$$

Since *W*_*a*_ is a tight frame satisfying *W*_*a*_^*H*^ *W*_*a*_ = *I*, letting *F* denote the full Fourier encoding matrix normalized such that *F* ^*H*^ *F* = *I*, we have

(7) Fukx,ky=Skx,ky,kx,ky∉Ω,Skx,ky+μS0kx,ky1+μ,kx,ky∈Ω,

where *S*_0_(*k*_*x*_, *k*_*y*_) = *FF*_*p*_^*H*^(*f* − *c* ^*k*^), *S*(*k*_*x*_, *k*_*y*_) = *FW*_*a*_^*H*^(*u*_*a*_^*k*^ − *d* ^*k*^), and *Ω* denotes the sampled K-space subset. In order to update *u*_*a*_ and *W*_*b*_, we introduce another assistant variable *v* = *W*_*b*_ *u*_*a*_ to decompose the coupling between *W*_*b*_ and *u*_*a*_ and therefore obtain the third-level Bregman iteration

(8) vk+1,Wbk+1=argminv,Wb⁡Wbuak−v+ek22+αv1,uak+1=argminua⁡ μWauk−ua+dk22+Wbkua−vk+1+ek22,ek+1=ek+Wbkuak−vk+1.

Similar to the update of *u*, we can easily get the least squares solution for *u*_*a*_

(9) $$\begin{array}{c} u_{a}^{k+1}=\frac{\mu \left(W_{a}u^{k}+d^{k}\right)+W_{b}^{H}\left(v^{k+1}-e^{k}\right)}{1+\mu }. \end{array}$$

As for the update of *v*, we temporarily fix the value of *W*_*b*_ and can easily obtain its update rule with the iterative shrinkage/thresholding algorithm (ISTA)

(10) $$\begin{array}{c} v^{k+1}=shrink\left(\;W_{b}u_{a}^{k+1}+e^{k},\frac{1}{\alpha }\;\right), \end{array}$$

where shrink(*x*, *a*) = sign⁡(*x*)max⁡(0, |*x*| − *a*). Now fix *v*, we update *W*_*b*_ by minimizing

(11) $$\begin{array}{c} \underset{W_{b}\in ⋀}{\mathrm{argmin}}{\left\|\;W_{b}u_{a}^{k}-v+e^{k}\;\right\|}_{2}^{2}. \end{array}$$

Instead of directly optimizing *W*_*b*_, we sequentially partition the coefficient vectors *v* − *e* into vectors and apply the technique of [16] to solve this subproblem using singular value decomposition (SVD), with the aim of learning its corresponding filter *b*_*m*_. To facilitate the readers to grasp the overall picture, we summarize the proposed TRIMS in Algorithm 1.

## 3. Experiments and Results

We evaluated the proposed method on three datasets, namely, a T1-weighted brain image obtained from GE 3T commercial scanner with an eight-channel head coil (TE = 11 ms, TR = 700 ms, FOV = 22 cm, and matrix = 256 × 256), a PD-weighted brain image scanned from 3T SIEMENS with an eight-channel head coil and MPRAGE (3D flash with IR prep, TE = 3.45 ms, TR = 2530 ms, TI = 1100 ms, flip angle = 7 deg., slice = 1, matrix = 256 × 256, slice thickness = 1.33 mm, FOV = 256 mm, and measurement = 1), and a physical phantom scanned from a 3T commercial scanner (SIEMENS MAGNETOM TrioTim syngo) with a four-channel head coil (TE = 12 ms, TR = 800 ms, FOV = 24.2 cm, and matrix = 256 × 256). Informed consent was obtained from the imaging subject in compliance with the Institutional Review Board policy. The Walsh adaptive combination method is applied to combine the multichannel data to a single-channel one corresponding to a complex-valued image. We have compared the proposed method to three state-of-the-art methods, namely, the representative analysis transform based DDTF-MRI, the synthesis dictionary based DLMRI, and the analysis-synthesis mixture based GradDLRec approach. TRIMS was implemented with shift invariant Haar wavelet filters for the fixed tight frame (the size of each filter is 2 × 2) and for initializing the second-level tight frame (the size of each filter is 4 × 4). The other three algorithms were implemented with their recommended parameter settings. To quantitatively evaluate the reconstruction accuracy of each method, we have employed peak signal-to-noise ratio (PSNR), relative error, and structural similarity (SSIM) index [18] which are defined as follows:

(12) PSNR=20 log10⁡max⁡u0Qu0−u^2,err⁡=u0−u^2u02,SSIM=lu^,u0α·cu^,u0β·su^,u0γ,

where SSIM is multiplicative combination of the three terms, namely, the luminance term $l(\hat{u},u_{0})$, the contrast term $c(\hat{u},u_{0})$, and the structural term $s(\hat{u},u_{0})$.

**Figure 1:**
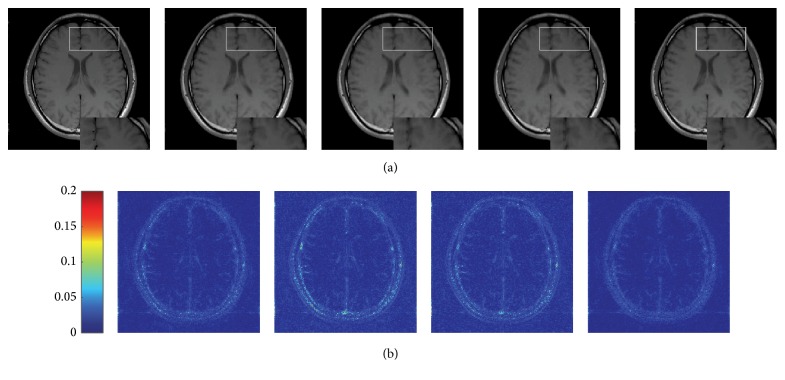
Visual quality comparison on GE MR images reconstructed by the four approaches from radially undersampled K-space data (25.16%). (a) From left to right: ground truth image and images reconstructed by the DDTF, DLMRI, GradDLRec, and proposed TRIMS; each one has an enlarged region for a closer comparison. (b) From left to right: color axis and difference images of the DDTF, DLMRI, GradDLRec, and TRIMS.

We firstly applied the four approaches to reconstruct T1-weighted MR image under the radial sampling scheme with the acceleration factor *R* = 4 (sampling ratio 25.16%). The reconstructed image obtained by each algorithm and the absolute difference between the reconstructed image and the ground truth image were displayed in Figure 1. We also present an enlargement area to reveal the fine details and structures each method has preserved. We can see that there exist somewhat blurring artifacts on the edges in the results reconstructed by the four methods. However, TRIMS can reconstruct an image closer to the one reconstructed from the full data. The absolute difference maps also indicate that TRIMS incurs less errors while reconstructing the MR image compared to the other three approaches.

**Figure 2:**
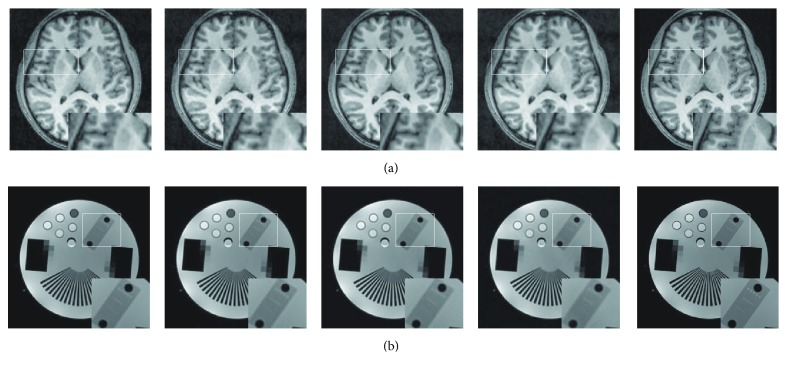
Visual quality comparison on PD-weighted and physical phantom MR images reconstructed by the four approaches from 2D randomly undersampled K-space data (9.312%). From left to right: ground truth image and images reconstructed by the DDTF, DLMRI, GradDLRec, and proposed TRIMS; each one has an enlarged region for a closer comparison.

We further utilized the four approaches to reconstruct the PD-weighted brain image from 9.13% of 2D randomly sampled K-space data.  Figure 2(a) displays the original image and the images reconstructed by the four approaches. For a close-up look, the white box enclosed part has been zoomed and presented at the right corner of the image. It can be observed that our method has produced an image closer to the original image. The four approaches were also evaluated on a scanned physical phantom which consists of quite a few regular structures with fine details.  Figure 2(b) provided the visual comparison results of the phantoms reconstructed from 12.79% of 2D randomly sampled K-space data. An area with different scales of lines was enlarged in each image to visualize the reconstruction accuracy of each method. It can be observed that the enlarged parts in the reconstruction results suffer from blur. Nevertheless, the proposed method can still produce an image with less blurry artifacts.

**Figure 3:**
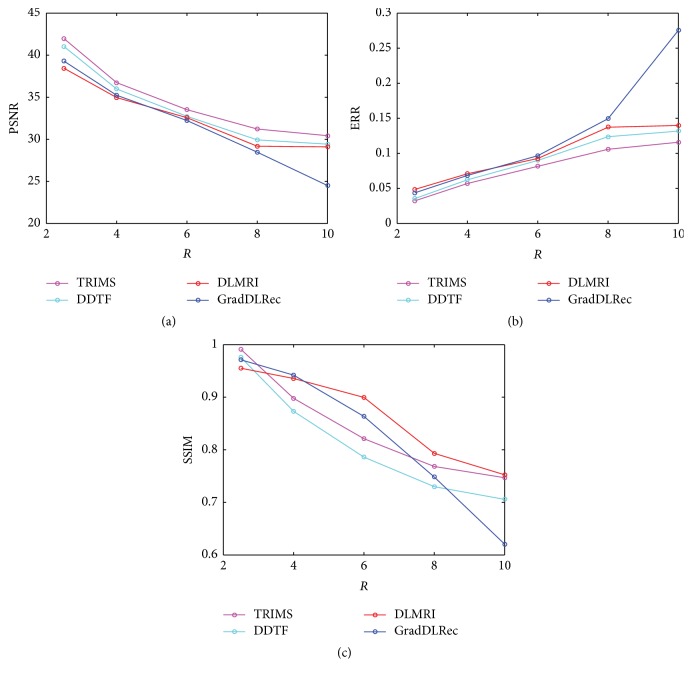
The average reconstruction errors in PSNR, relative error, and SSIM over all images with respect to different acceleration rates.

To test the sensitivity of the four methods to acceleration factors, we retrospectively undersampled the full K-space data with the 2D variable density scheme at 2.5-, 4-, 6-, 8-, and 10-time acceleration and employed the four methods to reconstruct MR images from the undersampled data.  Figure 3 has presented the average PSNR, relative error values, and SSIM over all the three images reconstructed by the four methods versus different acceleration factors. The two PSNR and relative error plots have demonstrated that the proposed method could achieve better reconstruction results at all acceleration rates. Nevertheless, we should admit that the plot of SSIM indicates that the proposed method does not produce the best results at all undersampling factors on average since the current tight frame size is relatively small based on the concern of the computational complexity. Better results can be produced if the size of the tight frame is set a little bigger.

**Figure 4:**
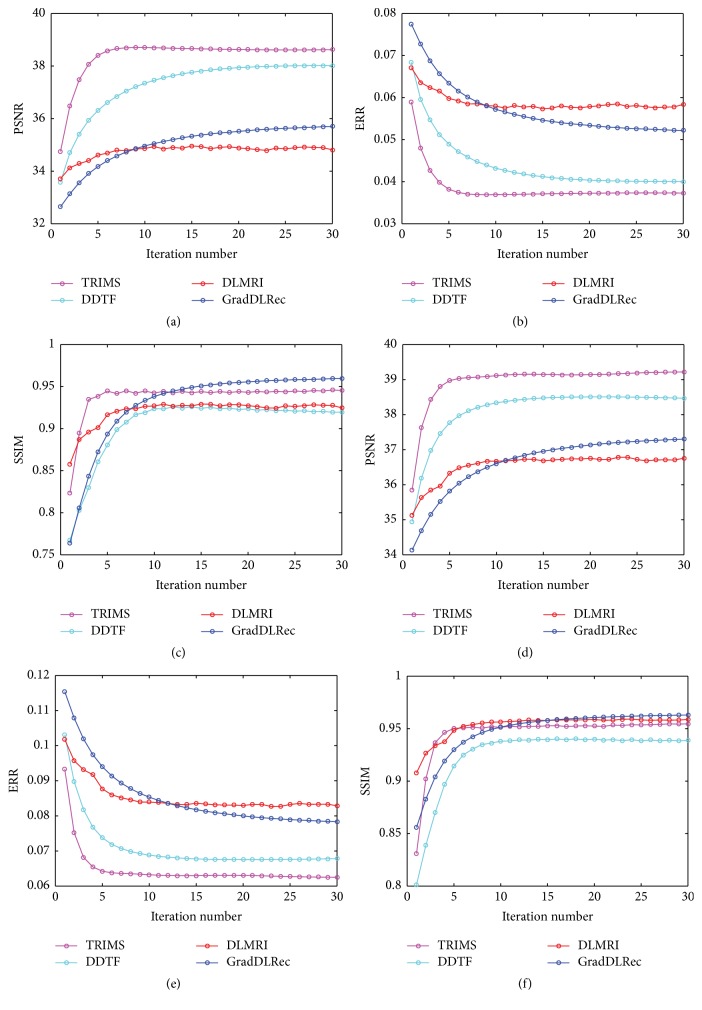
The convergence development of the four methods over acceleration rates 2.5 and 6 in PSNR, relative error, and SSIM while reconstructing the T1-weighted image.

We also have provided a comparison of the convergence property of the four methods over acceleration rates 2.5 and 6 on the T1-weighted image in Figure 4. As can be seen, the four methods all have approximately converged.

Finally, we compare the computational time of the four methods, which were implemented on a Windows 7 (64-bit) operating system equipped with 8 GB RAM and Intel® Core*™* i7-4770 CPU @ 3.40 GHz in MATLAB 2015a.  Table 1 lists the computational time for each method over the six acceleration rates. We can observe that TRIMS is more efficient compared to DLMRI and GradDLRec. It is even more efficient than DDTF since DDTF needs to train 64 filters, each size of which is 8 × 8, while TRIMS only needs to train 16 filters whose size is 4 × 4. Furthermore, it is worth mentioning that although the size of the to-be-learned tight frame of TRIMS is smaller than that of DDTF, the two-layer sparsifying nature has facilitated TRIMS to achieve better reconstruction results in shorter time compared to DDTF.

## 4. Conclusions

This paper proposes a two-layer tight frame sparsifying model, namely, TRIMS, for compressed sensing magnetic resonance imaging. This approach explores the strength of adaptive learning technique and tight frames for accurate reconstruction of MR images from undersampled K-space data. The experimental results demonstrated that the proposed TRIMS could accurately reconstruct MR images from a variety of undersampled data with proper efficiency.

## Figures and Tables

**Algorithm 1:**
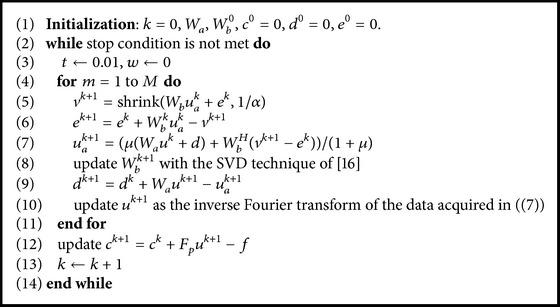
Reconstructing MR images from undersampled K-space data with TRIMS.

**Table 1:** The computational time (in second) comparison over different acceleration rates.

Image	PD-weighted image					
Acceleration rate	2.5	4	6	8	10	20
TRIMS	137	139	140	139	137	137
DDTF	148	149	148	148	148	148
DLMRI	1294	1234	1215	1205	1188	1161
GradDLRec	2644	2475	2386	2352	2338	2298
